# Acknowledgment to the Reviewers of *Audiology Research* in 2022

**DOI:** 10.3390/audiolres13010009

**Published:** 2023-01-19

**Authors:** Audiology Research Editorial Office

**Affiliations:** MDPI AG, St. Alban-Anlage 66, 4052 Basel, Switzerland

High-quality academic publishing is built on rigorous peer review. *Audiology Research* was able to uphold its high standards for published papers due to the outstanding efforts of our reviewers. Thanks to the efforts of our reviewers in 2022, the median time to first decision was 20.5 days and the median time to publication was 46.5 days. Regardless of whether the articles they examined were ultimately published, the editors would like to express their appreciation and thank the following reviewers for the time and dedication that they have shown *Audiology Research*:
Aazh, HashirKillan, EdwardAltoe, AlessandroKoning, Henk MariaArjmandi, MeisamKonopka, WieslawAttias, JosephKožuh, InesAuletta, GennaroKuthubutheen, JafriBakay, Warren M.H.Mancini, PatriziaBalzanelli, CristianoManzari, LeonardoBattelino, SabaMarcovich, IrinaBester, ChristoferMartellucci, SalvatoreBezdjian, ArenMartini, AlessandroBinos, ParisMartins, JorgeBlanchet, CatherineMarx, MathieuBoecking, BenjaminMasalski, MarcinBojić, TijanaMaslin, MikeBorkowski, PawełMatusiak, MonikaBrkic, Faris F.McCullagh, Marjorie C.Brotto, DavideMerchán, MIguel ÁngelBruschini, LucaMessina, AldoCalifano, LuigiMiyamoto, YukiCardon, EmilieMoulin, AnnieCasani, Augusto PietroMucci, VivianaCastellucci, AndreaMulsow, JasonČerný, RudolfMuralimanohar, Ramesh KumarCherchi, MarcelloMurofushi, ToshihisaChoung, Yun-HoonMurri, AlessandraChung, JaehoNeri, GiampieroCiodaro, FrancescoNi, AoxinCiorba, AndreaNie, KaibaoCiuman, RaphaelNishimura, KojiCoffin, Allison B.Nishiyama, TakanoriComacchio, FrancescoNoij, Kimberley S.Cywka, Katarzyna BeataObrist, DominikDa Cruz Fernandes, LucieneObrycka, AnitaDanesh, Ali A.Oron, YahavDe Laat, Jan A. P. M.Palma, SilviaDepireux, DidierParnes, Steven M.Deroche, Mickael L. D.Perez-Fernandez, NicolasDi Stefano, VincenzoPiras, GianlucaEduardo, Martin-SanzRajamani, SanthoshEdyta, PiłkaRalli, GiovanniEspinosa-Sanchez, Juan M.Rambold, Holger A.Etournay, RaphaëlRedaelli De Zinis, Luca OscarFabre, ChristolRiazuddin, SheikhFalcioni, MaurizioRichard, Craig A. H.Fioretti, AlessandraRöesch, SebastianFostick, LeahRogić Vidaković, MajaFranz, BurkhardRusinek, RafalFredianelli, LucaSacchini, SimonaFüllgrabe, ChristianSanchez-Lopez, RaulGabr, TakwaSatu, Turunen-TaheriGarbaruk, EkaterinaShoushtarian, MehrnazGarinis, Angela C.Skarzynska, MagdalenaGeal-Dor, MiriamSkarzynski, Piotr HenrykGéléoc, Gwenaëlle S. G.Specchia, AntonioGica, NicolaeStefania, GoncalvesGrilo, MargaridaSterkers, OlivierGummer, Anthony W.Strenzke, NicolaGuo, HongsunSvetlana, ChibisovaHahn, ChipSzczepek, Agnieszka J.Hoetink, Alexander E.Tamulevičius, GintautasHofer, JohannesTavartkiladze, GeorgeHorhat, DeliaTeggi, RobertoHosoya, MakotoTsetsos, NikolaosImai, TakaoUmat, CilaIndovina, IoleWaissbluth, SofiaIvanchenko, Maryna V.Walkowiak, AdamJedrzejczak, WiktorWang, WuqingJeong, Jun HuiWarnecke, MichaelaJiménez, EstíbalizWelch, DavidJohn, FerraroWest, NielsKamogashira, TeruWit, Hero P.Katrakazas, PanagiotisZakaria, Mohd NormaniKattah, Jorge C.Zhang, Yang

